# Integrated Analysis of Copy Number Variation and Genome-Wide Expression Profiling in Colorectal Cancer Tissues

**DOI:** 10.1371/journal.pone.0092553

**Published:** 2014-04-02

**Authors:** Nur Zarina Ali Hassan, Norfilza Mohd Mokhtar, Teow Kok Sin, Isa Mohamed Rose, Ismail Sagap, Roslan Harun, Rahman Jamal

**Affiliations:** 1 UKM Medical Molecular Biology Institute, Universiti Kebangsaan Malaysia, Cheras, Kuala Lumpur, Malaysia; 2 Department of Pathology, Faculty of Medicine, Universiti Kebangsaan Malaysia, Kuala Lumpur, Malaysia; 3 Department of Surgery, Faculty of Medicine, Universiti Kebangsaan Malaysia, Kuala Lumpur, Malaysia; 4 Department of Physiology, Faculty of Medicine, Universiti Kebangsaan Malaysia, Kuala Lumpur, Malaysia; 5 Department of Medicine, Faculty of Medicine, Universiti Kebangsaan Malaysia, Kuala Lumpur, Malaysia; Howard University, United States of America

## Abstract

Integrative analyses of multiple genomic datasets for selected samples can provide better insight into the overall data and can enhance our knowledge of cancer. The objective of this study was to elucidate the association between copy number variation (CNV) and gene expression in colorectal cancer (CRC) samples and their corresponding non-cancerous tissues. Sixty-four paired CRC samples from the same patients were subjected to CNV profiling using the Illumina HumanOmni1-Quad assay, and validation was performed using multiplex ligation probe amplification method. Genome-wide expression profiling was performed on 15 paired samples from the same group of patients using the Affymetrix Human Gene 1.0 ST array. Significant genes obtained from both array results were then overlapped. To identify molecular pathways, the data were mapped to the KEGG database. Whole genome CNV analysis that compared primary tumor and non-cancerous epithelium revealed gains in 1638 genes and losses in 36 genes. Significant gains were mostly found in chromosome 20 at position 20q12 with a frequency of 45.31% in tumor samples. Examples of genes that were associated at this cytoband were *PTPRT*, *EMILIN3* and *CHD6*. The highest number of losses was detected at chromosome 8, position 8p23.2 with 17.19% occurrence in all tumor samples. Among the genes found at this cytoband were *CSMD1* and *DLC1*. Genome-wide expression profiling showed 709 genes to be up-regulated and 699 genes to be down-regulated in CRC compared to non-cancerous samples. Integration of these two datasets identified 56 overlapping genes, which were located in chromosomes 8, 20 and 22. MLPA confirmed that the CRC samples had the highest gains in chromosome 20 compared to the reference samples. Interpretation of the CNV data in the context of the transcriptome via integrative analyses may provide more in-depth knowledge of the genomic landscape of CRC.

## Introduction

Colorectal cancer is a major health concern, with more than a million individuals diagnosed every year worldwide [1]. This cancer is among the top three of all cancers that lead to death worldwide [2]. In Malaysia, it ranks as the second most common cancer in both sexes [3].

One form of genetic instability that is observed in at least 85% of sporadic CRC cases is chromosomal instability (CIN) [4]. Aneuploidy is a consequence of CIN that leads to the gain or loss of whole or parts of chromosomal regions [5], and it may cause structural complexity that leads to genomic instability. One common form of structural variants due to CIN is known as copy number variations (CNVs), which is defined as a gain or loss of copies of DNA segments that are larger than 1 kb in length when compared to a reference genome [6]. CNVs can affect gene expression and have been associated with disease susceptibility. It has been suggested that transcriptional changes correspond to CNVs and alterations in gene dosage can be correlated with changes in expression level [7].

Thousands of CNV sites have been documented using microarray technology. Previous studies on colorectal cancers have revealed gains at chromosome 8q, 13 and 20q and losses at chromosome 8p, 17p and 18q [8], [9], [10], [11], [12]. These aberrations lead to the deletion or amplification of tumor suppressor genes, oncogenes, or non-coding RNAs such as miRNAs, which result in aberrant expression of genes that affect cancer-related biological processes [13], [14].

A gene that has one duplicated allele (a copy number of 3) has a higher level of expression than the wild-type [15]. Conversely, a gene that has one allele deleted (a copy number of 1) will have a lower level of expression [15]. Integrative analyses in CRC showed that expression levels of certain oncogenes and tumor suppressor genes were related to CNV [16]. For example, amplification or gain of the *MYC* gene at position 8q24 results in over-expression of this gene in CRC. Furthermore, deletion or loss of the *APC* gene at position 5q21 leads to its deregulated expression in CRC [17]. Similar patterns of correlation have also been observed in breast and lung cancers. Approximately 12% of changes in gene expression levels were reported to be in concordance with copy number in breast cancer [18], and approximately 78% genes showed a positive correlation between CNV and gene expression level in a lung cancer study [19].

The goal of this study was to obtain an insight into the molecular mechanisms of CRC via the analyses of CNV and genome-wide expression profiling of a primary tumor and its corresponding non-cancerous colonic epithelium. We also wanted to identify the relationship between the CNV profile and the transcriptome in CRC.

## Materials and Methods

### Patient recruitment

The study was performed with approval from the ethics committee of Universiti Kebangsaan Malaysia (UKM 1.5.3.5/244/UMBI-004-2012), and written informed consent was taken from each of the 64 patients who underwent surgery. None of the patients had received chemotherapy or radiotherapy treatment prior to surgery. Primary tumor and non-cancerous tissues (10 cm away from the tumor) were obtained immediately after surgery and snap-frozen in liquid nitrogen for storage.

### Genomic DNA and RNA isolation

Frozen sectioning was performed on all samples using a cutting thickness of 5 to 7 µm. The sections were mounted onto glass slides and stained with hematoxylin and eosin (H&E). The stained slides were then evaluated by a histopathologist to confirm the presence (>80% cancer cells) or absence of tumor cells and their corresponding non-cancerous cells. DNA was isolated using the Qiagen DNeasy Blood and Tissue Kit according to the manufacturer's protocol. Total RNA was extracted from 15 paired samples using the Qiagen QIAmp Mini Plus Kit. The quality of the isolated DNA and RNA was quantified using a NanoDrop ND-1000 spectrophotometer (Thermo Fisher Scientific, Waltham, MA), and only samples with a purity of 1.8 to 2.1 (A260/A280) were selected. The integrity of the isolated DNA samples was evaluated on 1.0% agarose gels, and the integrity of the isolated RNA was determined using a Bioanalyzer (Agilent Technologies, CA, USA). Samples with a RNA Integrity Number (RIN) of 6.0 and above were selected for gene expression profiling.

### CNV and gene expression profiling

All 64 paired samples were assayed using the Illumina HumanOmni1-Quad Bead Chip, which contains 1,140,419 single nucleotide polymorphism (SNP) loci, based on the Illumina Infinium II assay protocol (Illumina, San Diego, CA, USA). Gene expression profiling was performed on the 15 paired samples using the Affymetrix GeneChip Human Gene 1.0 ST array, which contains 36,079 transcripts with 28,869 well-annotated genes (Affymetrix Inc., Santa Clara, CA, USA). The RNA was prepared using the Applause WT-Amp ST System protocol before the hybridization process (NuGen Technologies Inc., San Carlos, CA, USA). The arrays were stained and scanned based on the GeneChip Whole Transcript (WT) Sense Target Labeling Assay protocol that was outlined by Affymetrix.

### Submission of microarray data to the ArrayExpress database

The raw and normalized microarray data were loaded into the ArrayExpress database: http://www.ebi.ac.uk/arrayexpress. The ArrayExpress accession is E-MEXP-3980.

### Statistical analysis of CNV profiling

The binary files (.*idat*) that were produced by the Illumina scanning software (Bead Scan Array Reader) were analyzed using the Illumina Genome Studio Genotyping Module version 3.2.33 (Illumina, San Diego, CA, USA) to obtain normalized genotype data. The genotype call rate threshold was set at ≥90%, and the final report of the normalized genotype data was transferred to a third-party program, Partek Genomic Suite version 6.6 (Partek Inc., St. Louis, MO, USA), to determine the CNV profiles.

**Figure 1:**
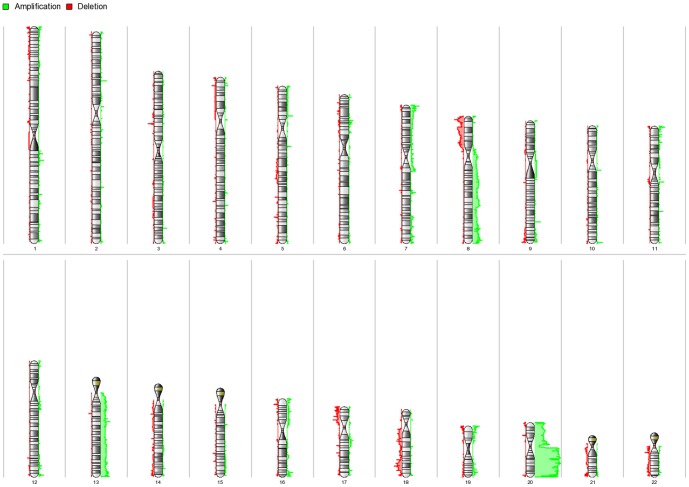
Karyogram view of detected gains and losses regions across autosomes. Gains are shown in green and losses are shown in red. The length of the horizontal bar corresponds to number of samples involved at the respective cytoband. Most of the gains were found at the long arm of chromosome 20 and losses were mainly observed at the short arm of chromosome 8.

Paired CNV analysis was carried out by comparing the intensity of each hybridization signal from a tumor sample to that of its matched non-cancerous epithelium. The genomic segmentation algorithm was used to detect CNV gains and losses. The following stringent parameters were set to reduce any false-positive alteration: each segment must contain a minimum of 10 consecutive filtered probe sets, a p-value threshold of 0.001 when compared to the neighboring adjacent regions and a signal-to-noise threshold of 0.5. The cut-off value for the gain was set at above 2.3, while loss was set at below 1.7. CNV was called for the gains or losses that occurred in at least 10% (7 samples) of the total samples. A full listing of all CNV gains and losses are included in Table S1. The chromosomal locations of the copy number gains and losses of the 22 autosomes are shown by karyograms (Figure 1).

### Statistical analysis of gene expression profiling

**Figure 2:**
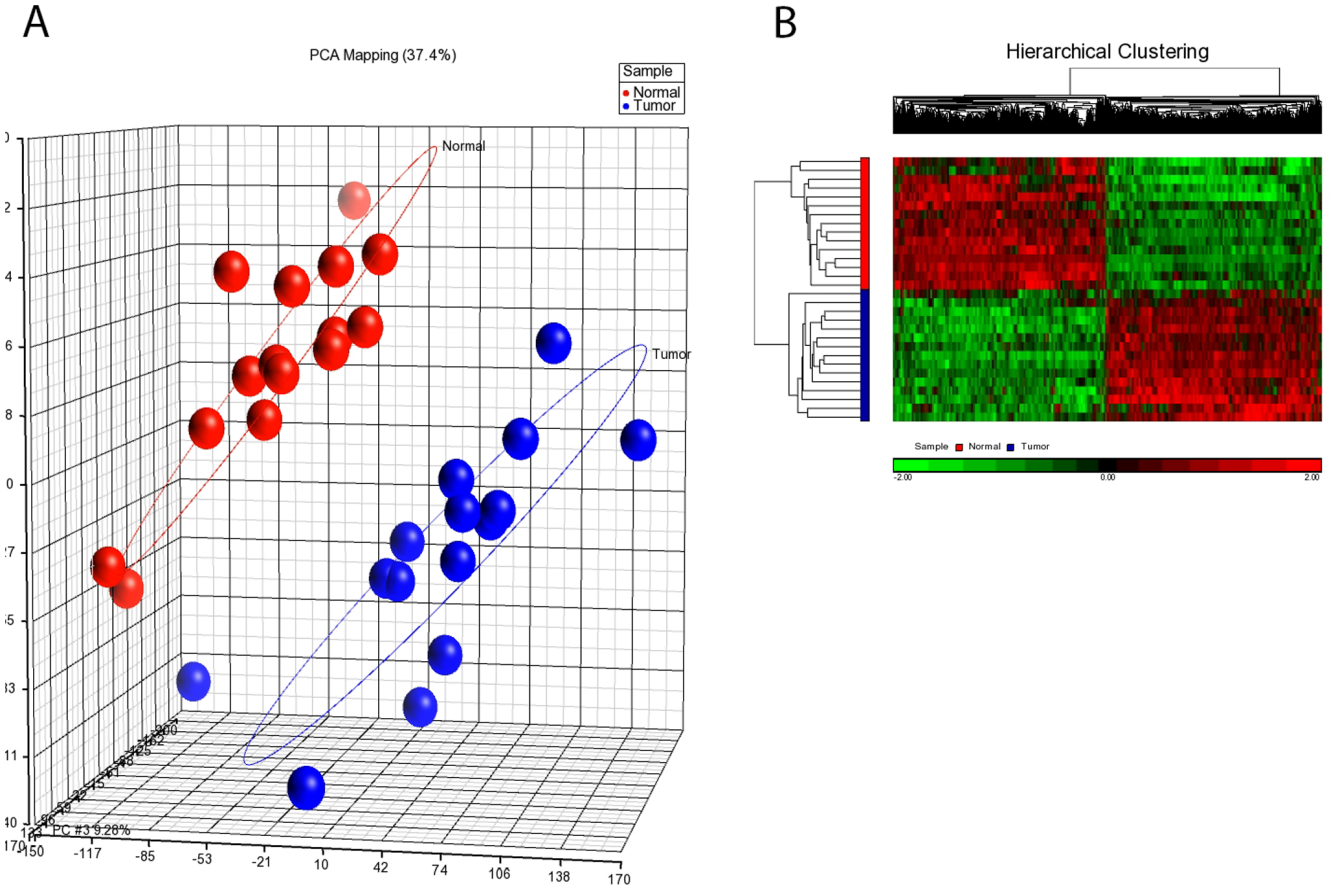
Plots of principal components analysis (PCA) and hierarchical clustering of gene expression datasets. (A) PCA scatter plot of CRC data. Each point represents sample. Points are colored by group status with blue representing non-cancerous epithelium and red representing tumor tissue. (B) Hierarchical clustering of mRNA profiles. Samples are indicated along the horizontal axis and grouped by the color bar between the dendogram and the heat map. Blue represents non-cancerous epithelium and red represents tumor tissue. Overall, there was a clear separation between non-cancerous epithelium and tumor tissue group when examined by both PCA (Figure 2A) and hierarchical clustering (Figure 2B).

The Affymetrix CEL files were imported to Partek Genomic Suite 6.6 (Partek Inc., St. Louis, MO, USA) to perform gene expression profiling analysis. Raw CEL files were processed for background correction and quantile normalization (median scaling) using the robust multi-array averaging (RMA) method. A principal component analysis (PCA) plot was generated as the quality control step (Figure 2A), and the batch effect was removed as a source of variation. A three-way ANOVA was performed across all samples. Statistically significantly expressed genes were identified using the mixed model analysis of variance with a false discovery rate (Benjamini–Hochberg test) adjusted p value of ≤0.05 and fold-change values of −2 to 2. Hierarchical clustering was generated to visualize patterns of expression in the data (Figure 2B). Gene ontology enrichment analysis was performed using DAVID (Database for Annotation, Visualization and Integration Discovery) Bioinformatic tools.

### Integration of copy number variation and genome-wide expression analyses

**Figure 3:**
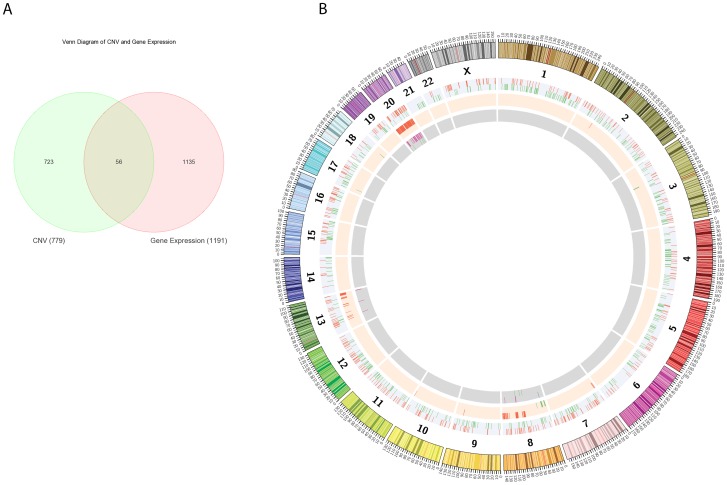
Overlapped genes of integrated CRC datasets. (A) Venn-diagram representing the common genes in CNV and gene expression datasets revealed 56 overlapping genes. (B) Circular map showing overview of CNVs and gene expression data. Chromosomes are shown in the color coded of the outer most ring. The second ring shows the distribution of gene expression profile. (red indicates up-regulated genes and green indicates down-regulated genes). The inner ring represents CN changes (red denotes gain in CN and green denotes loss in CN). The innermost ring shows the distribution of the two overlapping datasets.

Data from CNV and genome-wide expression analyses were analyzed individually. To identify the significant genes that exhibited CNV and gene expression changes, we overlapped the two datasets as presented in the Venn diagram (Figure 3A). The chromosomal locations of the overlapping genes between the two datasets are shown in a circular map (Figure 3B).

### Sub-analysis of the data from the copy number variation and genome-wide expression microarray for the 15 paired cases

**Figure 4:**
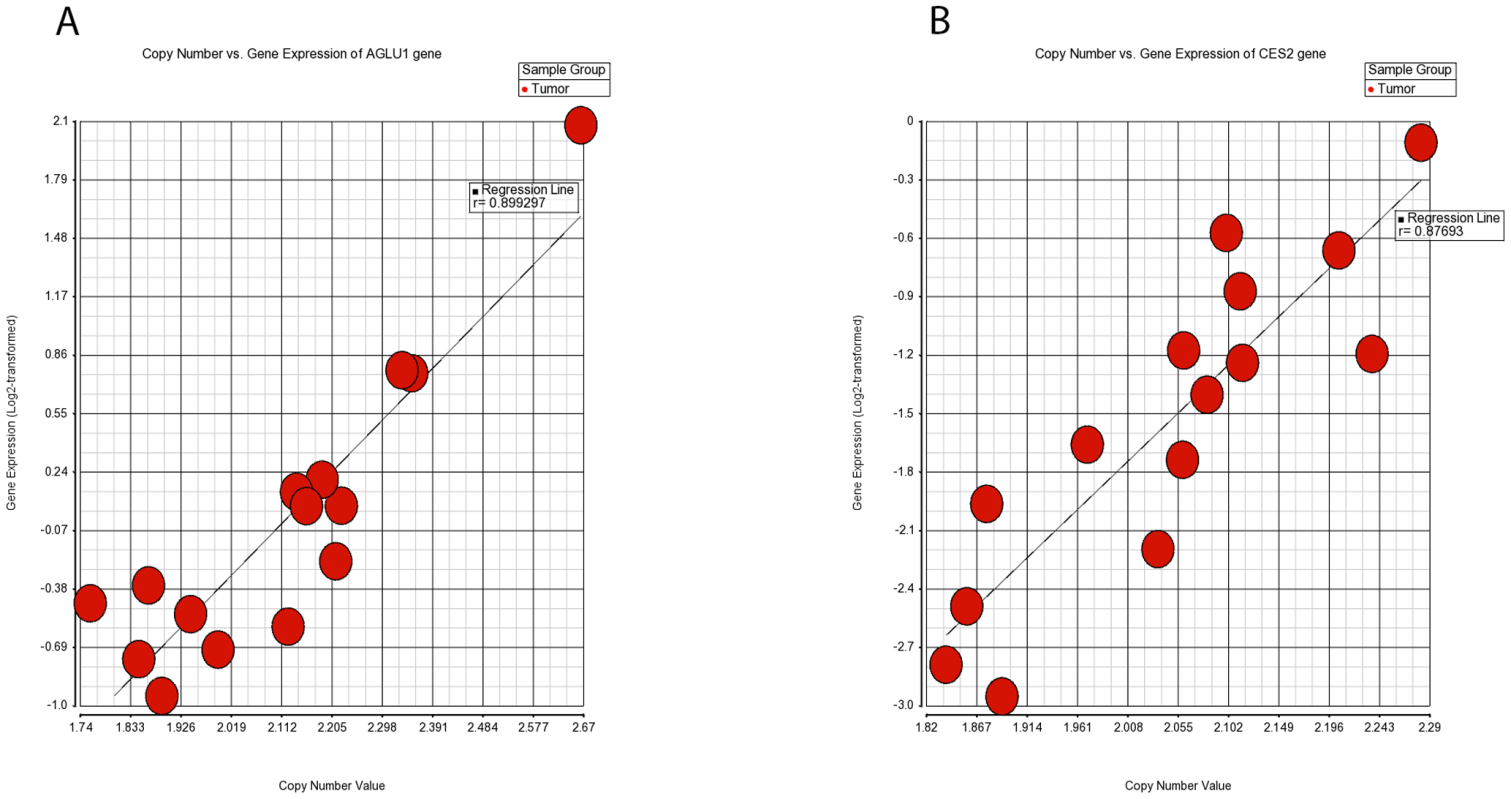
Correlation of gene expression and CNV datasets in 15 paired subsets of CRC patients. Scatter plots of gene expression (y-axis) correlating to copy number (x-axis) with differential expression & CN change in CRC for *ARGLU1* (Figure 4A) and *UGGT2* (Figure 4B) genes. Each dot represents one sample.

We analyzed the data that were obtained from copy number and genome-wide expression profiling of the 15 paired samples using Partek Genomic Suite 6.6 (Partek Inc., St. Louis, MO, USA). The genomic segmentation algorithm was applied with the parameters that were mentioned in the copy number analysis section. The resulting spreadsheet contained individual markers that displayed the aberrant levels of DNA in each tumor relative to its paired normal. Expression data were normalized to the baseline and the ratios were log2-transformed prior to analysis. Both datasets were correlated using Pearson's linear correlation method and we generated a scatter plot for viewing the results (Figure 4A and 4B).

### Multiplex Ligation Probe Amplification (MLPA)

To validate the CNV profiling results, we selected chromosome 20 for the MLPA assay using the SALSA MLPA P157 20q probe mix according to the manufacturer's protocol (MRC-Holland, Amsterdam, and The Netherlands). The probe mix contains 34 probes for 26 different genes that are located on 20q. Fragment analysis of the PCR products was performed using the GeneScan-500 LIZ Size Standard on the Applied Biosystems 3130 DNA Analyzer (Applied Biosystems, Foster City, CA). The fluorescence data were collected during fragment separation and imported to the Coffalyser.Net software (MRC-Holland, Amsterdam, and The Netherlands) for analysis.

## Results

### Demographic Data

Of the 64 patients, 39 (60.94%) were females and 25 (39.06%) were males (Table 1). The majority were Malays (48.44%), followed by Chinese (46.88%) and Indians (4.69%). Based on the Dukes' classification, 33 patients (51.56%) were of Dukes' B, 27 patients (42.19%) were of Dukes' C and 4 patients (6.25%) were of Dukes' A. The mean age was 56±13.35 years old.

**Table 1:** Distribution of clinicopathological features among 64 paired CRC sample.

Characteristics	Number (n)	Percentage (%)
**Gender**		
Male	25	39.06
Female	39	60.94
**Age**		
<50 years old	11	17.19
>50 years old	53	82.81
**Race**		
Malay	31	48.44
Chinese	30	46.88
India	3	4.69
**Stage**		
Duke's A	4	6.25
Duke's B	33	51.56
Duke's C	27	42.19
**Differentiation**		
Well Differentiated	31	48.44
Moderately Differentiated	28	43.75
Poorly Differentiated	5	7.81
**Location**		
Right	15	23.43
Left	49	76.56

### Copy number gains were frequently found in the q arm of chromosome 20

Of the 8722 genomic segments in all samples, we narrowed down our analysis to 2212 CNV regions that corresponded to the autosomes that showed amplifications (Table S1). The CNVs were scattered across chromosome 1 to 22 with the highest amplification found in chromosome 20, which had 388 segments that represented 17.5% of all gains. The second highest number of gains was documented at chromosome 7, with 369 genomic segments, followed by chromosome 8, with 338 genomic segments. The longest length for the CNV gain segments was between 8q22.1 and 8q22.2 corresponding to 4,070,488 bp. A total of 1,638 genes were amplified in all samples and 765 of these were unique or non-redundant genes. Cytoband 20q12 had the highest frequency of gains involving at least 45.31% of the tumor samples. Eight genes were identified at this locus and three of them, namely *PTPRT*, *CHD6* and *EMILIN3*, have been previously described in colorectal cancer. Figure 1 shows the distribution of these genes at chromosome 20, with gains shown in green. Details on the top 10 significant genes according to the RefSeq database are provided in Table 2.

**Table 2:** Top 10 significant copy number gain regions.

Cytoband	Genes	Frequency (%)	Start	Stop	Length (bps)	Gain value
20q12	*PTPRT*	45.31	40848490	40858418	9929	2.50
20q12	*CHD6, EMILIN3, LPIN3, PLCG1, TOP1, ZHX3, MAFB*	43.75	39344635	41684451	2339816	2.50
20q13.12	*EYA2, MIR3616, LOC100131496, ZMYND8*	43.75	45757009	45948813	191805	2.49
20q13.13	*LOC284749, PREX1, CSE1L, KCNB1, SNAI1, SATA2, STAU1*	43.75	46984676	48922757	1938081	2.49
20q11.23	*BLCAP, CTNNBL1, RBL1, SRC, MANBAL*	43.75	35626170	36460629	834460	2.48
20q11.21	*DEFB115, DEFB116, DEFB118, DEFB119, DEFB121, DEFB122, FRG1B*	42.19	29466797	30046837	580040	2.50
20q11.23 – 12	*ACTR5, ADIG, DHX35, TGM2, VSTM2L*	42.19	36484397	39212610	2728214	2.49
20q13.11–13.12	*IFT2, L3MBTL1, MYBL2, SGK2, SRSF6*	42.19	41917787	42308263	390477	2.51
20q11.21	*BCL2L1, COX412, DEFB124, HM13, ID1*	42.19	30049554	30310687	261134	2.50
20q11.22	*AHCY, DYNLRB1, MYH7B, ITCH, NCOA6*	40.63	32766406	33610899	844493	2.49

### Copy number losses were mainly found in the p arm of chromosome 8

A deletion or loss of copy number value less than 1.7 was observed throughout the chromosome 1 to 22 autosomes, with the majority detected at chromosome 8, which had 225 CNV affected segments. The specific region for the losses was observed at position 8p23.3 with 17.9% occurrence in all tumor samples. The second-highest frequency of losses was detected in chromosome 17, with 213 genomic segments, followed by chromosome 19, with 184 genomic segments. The longest loss of a genomic segment was at 8p23.1 to 8p22, which consisted of 3,093,282 bp. We identified only 14 unique or non-redundant genes (Table 3). Analysis of the individual genes in chromosome 8 revealed that only 5 of the genes were previously reported to be related to CRC; these genes were *CSMD1*, *DLC1*, *TUSC3*, *SGCZ* and *LONRF1*. The distribution of the losses, shown in red, can be observed in the karyotype diagram as shown in Figure 1.

**Table 3:** The top most significant CN loss regions.

Cytoband	Genes	Frequency (%)	Start	Stop	Length (bps)	Loss value
8p23.2	*CSMD1*	17.18	4014635	4023153	8519	1.60
3p14.2	*FHIT*	11.0	60252758	60260269	7512	1.45
18q22.1	*LOC284294, LOC400654*	11.0	61918208	61919976	1769	1.63
17p12	*SHISA6*	11.0	11352448	11354727	2280	1.37
8p23.1–8p22	*DLC1, C8orf48, KIAA1456, LOC340357, LONRF1, TUSC3, MIR383, SGCZ*	11.0	12601480	15694761	3093282	1.65
8p12	*TEX15*	11.0	30675344	30698971	23628	1.59

### Analysis of gene expression profiling

Analysis revealed significant differences in gene expression between the primary tumor and non-cancerous epithelial samples. Classification by PCA showed a clear separation of two distinctive groups according to the tissue type (Figure 2A). A total of 1408 genes were differentially expressed by at least two-fold with statistical significance (p<0.05). However, a single gene is represented by multiple probe sets called ‘siblings probes’ in the Affymetrix GeneChip. Therefore, any redundant probe sets were filtered, which left 1191 unique genes for further analysis. Of these, 584 genes were found to be up-regulated while another 607 genes were found to be down-regulated. Most of the up-regulated genes were found in chromosomes 7 (n = 48, 8.22%), 2 (n = 44, 7.53%) and 12 (n = 42, 7.19%). Down-regulated genes were mainly located at chromosomes 1 (n = 73, 12%), 2 (n = 44, 7.25%), 3 and 4 (n = 43, 7.08%). Among the up-regulated genes were *MYC*, *CD44*, *ABC22*, *TIMP1*, and *BIRC5*, while the down-regulated genes included *FAS*, *KLF4*, *UGTIA1* and *CA2*. A full list of the differentially expressed genes and their corresponding fold-changes in expression and p values are provided in Table S2. Hierarchical clustering was generated and visualized via a heat map, where two distinctive expression patterns can be observed (Figure 2B). The functional characterization of the significant genes were performed using GO analysis, and they were found to be distributed throughout the top five classes, which included cell cycle, cell division, chromosome segregation, nucleoside binding and ATP binding (p<0.05).

### Effect of CNV on the expression of colorectal cancer-related genes

Integration of the two profiling datasets showed 56 overlapping genes (Figure 3A and 3B), and a full list of these overlapping genes is presented in Table S3. A total of 1,135 genes with only gene expression changes and 723 genes with CNV changes but no changes in transcript levels were observed. Integration of CNV and gene expression analyses showed a positive association in 48 genes (85.7%) and a negative association in the remaining 8 genes (14.3%) (Table 4).

**Table 4:** Categories of overlapping genes.

CNV	Gene Expression	No of overlapping genes
Gain	Up-regulated	47
Gain	Down-regulated	8
Loss	Up-regulated	0
Loss	Down-regulated	1
Total		**56**

To further understand further the biological function of these genes, the 56 genes were subjected to functional annotation and classification analysis using DAVID v6.7. We found the genes to be related to biological processes and cellular components.

**Figure 5:**
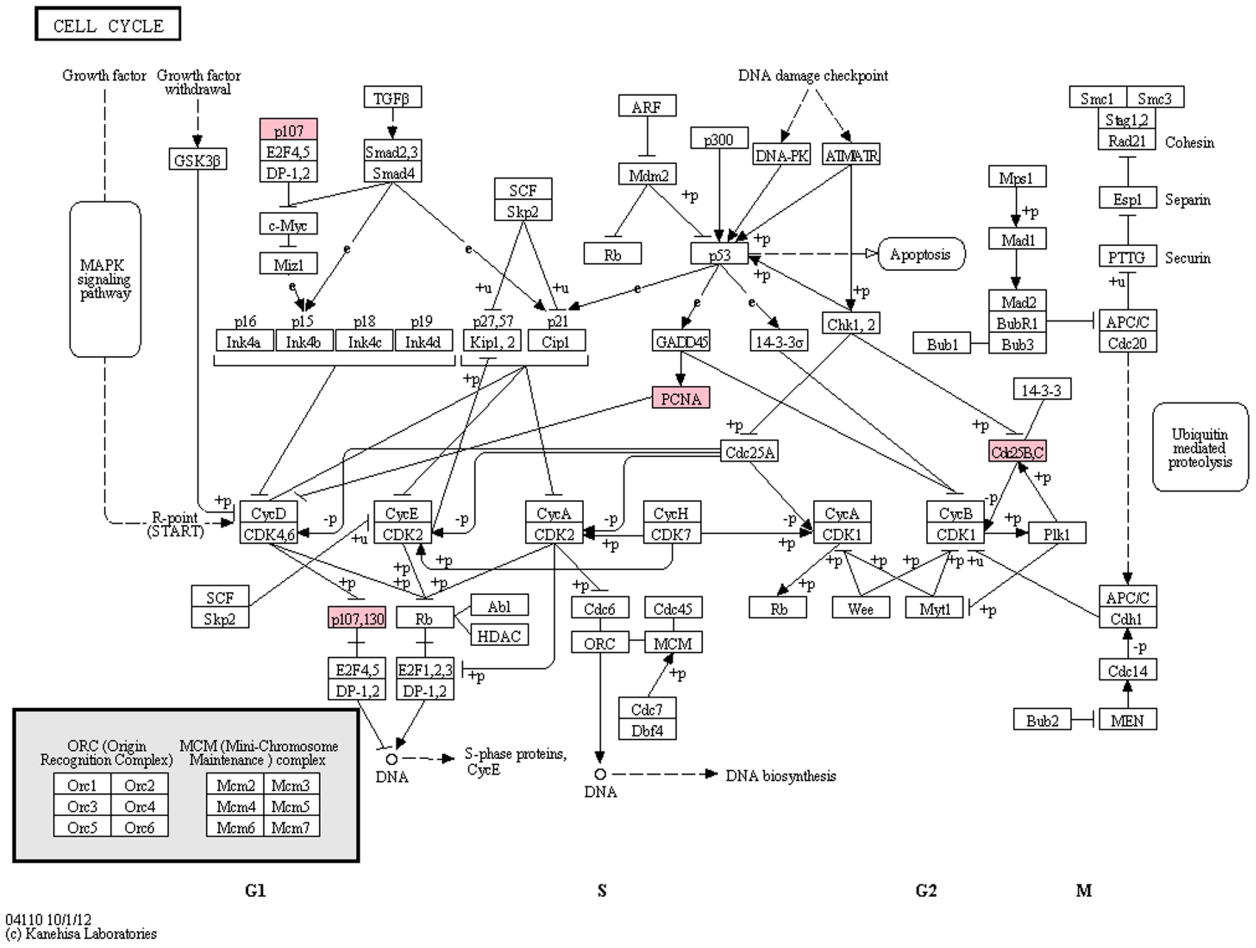
Cell cycle map from KEGG pathway. Cell cycle was found to be the most significant enriched pathway (p<0.05). Genes involved (*CDC25B, PCNA and p107/RBL1*) shown in red color box indicates the genes to have gain in CN and increased level of expression.

These genes were further mapped to the KEGG pathway database to determine the interactions of the candidate oncogenes or tumor suppressor genes that were identified by CNV and the expression array. The analysis revealed that the cell cycle was the most significantly enriched pathway and *CDC25B*, *PCNA* and *p107/RBL1* were the key, involved genes (Figure 5).

### Correlation of CNV with gene expression of colorectal cancer-related genes on a subset of 15 paired samples

To determine the relationship between DNA copy number and gene expression, we applied the Pearson's linear correlation model (Figure 4A & 4B). The correlation values were found between −0.85 to 0.89. Using a cut-off of p<0.05, 2159 genes showed significant correlations between copy number and gene expression. A total of 914 transcripts from 616 genes showed a good correlation (r>0.6) between copy number and gene expression. The top five-correlated genes were *ARGLU1*, *UGGT2*, *CES2*, *FUT10* and *PAOX*. A full list of the correlated genes with their Pearson's correlation and p values, can be viewed in Table S4.

### Validation of CNV profiling using MLPA

We performed the MLPA assay and targeted 26 genes in 150 samples (50 normal tissues and 100 tumors) to validate the CNV profiling data. We chose probes that covered the q arm of chromosome 20 and the results were considered acceptable if the control peak fell within the range of 0.8 to 1.2. A deletion was scored if the mean dosage of the test to the internal control peaks was less than 0.7, and duplication was scored if the mean dosage was 1.3 or greater. A normal reference sample showed no copy number alterations in any of the genes, and MLPA analysis showed that copy number gain was detected in all twenty-six tested genes. Table 5 summarizes the MLPA analysis with the mean values of each gene.

**Table 5:** Validation of copy number profiling by MLPA.

Gene	Frequency (%)	Mean±SD
BCL2L1	27	1.61±0.51
TPX2	16	1.43±0.45
HCK	31	1.62±0.52
DNMT3B	32	1.53±0.47
E2F1	16	1.50±0.50
NCAO6	41	1.58±0.54
SRC	21	1.64±0.54
NNAT	25	1.44±0.44
TOP1	36	1.50±0.49
MYBL2	24	1.54±0.51
HNF4A	25	1.50±0.51
UEB2C	18	1.55±0.52
MMP9	24	1.49±0.50
CSE1L	16	1.41±0.42
PTPN1	42	1.50±0.43
NFATC2	30	1.47±0.42
ZNF217	39	1.47±0.56
BCAS1	36	1.46±0.46
AURKA	38	1.47±0.46
STX16	29	1.47±0.44
GNAS	22	1.35±0.40
ADRM1	20	1.60±0.55
KCNQ2	17	1.55±0.50
EEF1A2	19	1.60±0.57
TNFRSF6B	6	1.67±0.60
OPRL1	24	1.57±0.51

## Discussion

We performed an integrated analysis using multiple datasets in colorectal tissues to identify the differentially expressed genes with alteration in genomic segments. We analyzed the CNVs and gene expression profiling in 64 paired and 15 paired CRC tissues respectively. The use of adjacent non-cancerous tissues from the same individual reduced the variations that were caused by inter-individual heterogeneity.

The present study identified a number of focal genomic gains and losses in CRC, which showed some concordance with the results from previous studies [20], [21], [22]. Individual analysis of the CNV dataset revealed significant gains in the chromosome 20q that were also highly consistent with the previous studies [23], [24]. Similar gains had also been observed in breast cancer and primary gastric cancer [25]. Cytoband 20q12 showed the highest frequency of gains that spanned from 39239931 to 41684451 bp with the involvement of eight genes, including *PTPRT*, *CHD6*, *EMILIN3*, *LPIN3*, *PLCG1*, *TOP1*, *ZHX3* and *MAFB*. Of the eight genes, *TOP1*, *PLCG1* and *PTPRT* were related to CRC.

Topoisomerase 1 (*TOP1*) is an oncogene that catalyzes the unwinding of DNA and creates single-strand molecules, which are required in numerous biological processes such as DNA replication, transcription and DNA repair [26]. A study of *TOP1* using array CGH found gains in its copy number in CRC samples [9]. An increased copy number of *TOP1* was also detected in Stage III CRC patients with an average of four gene copies for every cell using a fluorescence *in situ* hybridization (FISH) method [27], [28].

The second gene that was related to CRC identified in this study was phospholipase C gamma 1 (*PLCG1*), which is a signaling molecule and is a neighboring gene of *TOP1*. *PLCG1* is activated in response to growth factor stimulation and is involved in the regulation of a variety of cellular functions such as cell migration, invasion and metastasis [29], [30], [31].

Protein tyrosine phosphatase receptor-T (*PTPRT*) is situated within the amplified region of 20q12 between 39344635 to 41684451 bp. It is a tumor suppressor gene and has been shown to play integral roles in cell adhesion and intracellular signaling [32]. Gain of copy number involving the *PTPRT* gene has been identified in a previous study on ovarian clear cell carcinoma by using array CGH [33]. The gain in copy number of this gene may be as a result of the ‘passenger gene’ effect because we could not detect any changes in its gene expression.

Other significantly amplified cytobands in the q arm of chromosome 20 encoded well-established oncogenes that were associated with CRC, and these included 20q11.21 (*BCL2L1*), 20q13.2–20q13.31 (*AURKA*), 20q11.23 (*SRC* & *CTNNBL1*), 20q11.21–20q11.22 (*DNMT3B*) and 20q11.22 (*DYNLRB1*). These findings suggest the important involvement of multiple candidate genes within the long arm of chromosome 20 in cancer development and progression. The MLPA appears to be a reliable and efficient method to evaluate DNA copy number changes because majority of these tested genes revealed concordance with the microarray results.

Copy number losses were mainly found at the p arm of chromosome 8. The highest loss was found at cytoband 8p23.2 from 4008230 to 4027339 bp. Among the genes that reside at this region is the CUB and Sushi Multiple Domains 1 gene (*CSMD1*), which is a tumor suppressor gene that codes for multiple domain complement regulatory and adhesion protein. Focal copy number loss of the *CSMD1* gene has been observed in CRC [34], breast cancer [35] and gastric cancer [36], and decreased level of *CSMD1* expression was reported to be significantly associated with high-tumor grade and reduced overall survival in a breast cancer study [37]. Another region that was noted to exhibit copy number loss that was identified in this study was cytoband 8p23.1–p22, that included a region that covered 12601480 to 15694761 bp. A gene that is located within this region is the Deleted in Liver Cancer 1 (*DLC1*), which was reported to be a tumor suppressor gene and has been shown to undergo copy number loss in several cancers, such as hepatocellular carcinoma and breast cancer [38], [39].

We did a sub-analysis of 15 paired samples of CRC to evaluate the relationship between copy number changes and gene expression. Genes involved in CRC, such as *MYC* (v-myc avian myelocytomatosis viral oncogene homolog) [40] *CCNB1* (cyclin B1) [41] and *PLK1* (polo-like kinase 1) [42], were up-regulated and concordantly amplified in copy number in our study. Seven genes were found to be down-regulated with loss in copy number but only two genes, *MUC17* (mucin 7) [43] and *CES* (carboxylesterase 2) [44] have been shown to be related to CRC.

Integrated analysis using a Venn diagram showed that 85.7% of genes had a positive association. When mapped to the KEGG pathway, the cell cycle was identified as the most significantly enriched pathway (p<0.05). The cell cycle is a critical regulator of cell proliferation, and growth and cell division after DNA damage. The cell cycle pathway is mainly driven by the cyclin-dependent kinase (CDKs) family and their regulatory subunits, the cyclins. The cell cycle has four phases and the two major checkpoints at the G1-S and G2-M transitions to maintain the correct order of events [45]. The loss of cell cycle checkpoint control promotes genetic instability, which leads to uncontrolled cell proliferation and could promote cancer development [46].

Cell division cycle 25B (*CDC25B*) is a member of the cell division cycle (CDC) phosphatase family that functions as activators of CDKs and cyclin complexes to regulate progression of the cell cycle [47]. *CDC25B* is responsible for the initial dephosphorylation and activation of the CDKs, thus initiating the sequence of events that leads to entry into mitosis [48], [49]. Over-expression of *CDC25B* has been observed in 43% of CRC patients and is correlated with poor prognosis [50]. Increased *CDC25B* level is sufficient to impair the DNA damage checkpoints, which in turn, increases spontaneous mutagenesis and interferes with the entry into mitosis [51], [52], [53].

Proliferating cell nuclear antigen (*PCNA*) was reported to be essential for DNA replication, DNA repair and cell cycle regulation [54]. Retinoblastoma-like 1 (*p107/RBL1*) is a member of the retinoblastoma gene family (RB), and the genes in this family have been identified as tumor suppressors. RBL1 and other RB proteins cooperate to regulate cell cycle progression through G1 phase of the cell cycle [55]. However, the mechanism of PCNA and RBL1 involvement in the cell cycle pathway of CRC is still unclear and requires further exploration.

We also found genes with negative associations between copy number and gene expression levels. The genes include *BCAS1*, *EDN3*, *FABP4*, *MATN2*, *SDCBP2*, *SPTLC3*, *TRPA1* and *WFDC2*. This paradox of a negative relationship between copy number status and gene expression has also been observed in a previous study on CRC [56] and might be attributed to the multiple mechanisms that are responsible for normal and abnormal control of gene expression, including those related to mutation, promoter methylation and miRNA expression [57]. To understand this phenomenon, an approach using deep sequencing technology will most likely probably provide an answer to these unexpected findings.

In conclusion, by integrating the datasets from two different profiling studies, we successfully identified 56 overlapping genes with changes in copy number and gene expression. The cell cycle was identified as the key signaling pathway from this integrated analysis. However, future studies are necessary to determine the impact of these genes on the outcome of the disease.

## Supporting Information

Table S1 **Full information on copy number variation profile of 64 colorectal cancer patients.**
(XLSX)

Table S2 **Full information on gene expression profile of 15 colorectal cancer patients.**
(XLSX)

Table S3 **Full list of 56 overlapped genes following integration analysis of both datasets.**
(XLSX)

Table S4 **Full list of sub-analysis between CNV and gene expression of 15 paired subset colorectal cancer patients.**
(XLSX)
